# Expiratory and Inspiratory Cries Detection Using Different Signals' Decomposition Techniques

**DOI:** 10.1016/j.jvoice.2016.05.015

**Published:** 2017-03

**Authors:** Lina Abou-Abbas, Chakib Tadj, Christian Gargour, Leila Montazeri

**Affiliations:** * Electrical Engineering Department, École de Technologie Supérieure, Montreal, Canada; † Electrical Engineering Department, Polytechnique Montreal, Canada

**Keywords:** automatic segmentation, empirical mode decomposition, wavelet packet transform, Gaussian mixture models, hidden Markov models

## Abstract

This paper addresses the problem of automatic cry signal segmentation for the purposes of infant cry analysis. The main goal is to automatically detect expiratory and inspiratory phases from recorded cry signals. The approach used in this paper is made up of three stages: signal decomposition, features extraction, and classification. In the first stage, short-time Fourier transform, empirical mode decomposition (EMD), and wavelet packet transform have been considered. In the second stage, various set of features have been extracted, and in the third stage, two supervised learning methods, Gaussian mixture models and hidden Markov models, with four and five states, have been discussed as well. The main goal of this work is to investigate the EMD performance and to compare it with the other standard decomposition techniques. A combination of two and three intrinsic mode functions (IMFs) that resulted from EMD has been used to represent cry signal. The performance of nine different segmentation systems has been evaluated. The experiments for each system have been repeated several times with different training and testing datasets, randomly chosen using a 10-fold cross-validation procedure. The lowest global classification error rates of around 8.9% and 11.06% have been achieved using a Gaussian mixture models classifier and a hidden Markov models classifier, respectively. Among all IMF combinations, the winner combination is IMF3+IMF4+IMF5.

## Introduction

Crying is the only possible way for newborns to express their needs and their physical conditions because they are not able to communicate with words. Cry signals have been studied for many years, and it has become evident that cry signals can provide valuable information concerning physiological and physical states of infants. Most research on infant cry focused on extracting information from infant cry signals with known medical problems such as prematurity asphyxia, hypoglycemia, Down syndrome, and meningitis. For example, the cries of infants with neonatal asphyxia and meningitis are high-pitched, and the cry duration is very short or unusually long with melody type rising or falling-rising in comparison with healthy infants. Preterm babies have higher minimum fundamental frequency than normal babies. Cries of infants with hyperbilirubinemia have significant changes in fundamental frequency over a 100-ms period. For the reason of cries, features such as pitch and loudness are able to distinguish hunger cry from pain cry.^1^, ^2^, ^3^, ^4^

Given these pieces of evidence, many researchers have suggested an automatic system to classify infant cries, which is more like a pattern recognition problem, similar to automatic speech recognition (ASR) systems. The aim of the automatic classification system is to give clinicians an early diagnostic result if a baby may have high probability to get specific types of medical diseases. As in any ASR system, a cry classification system needs a segmentation module that can detect useful parts of recorded signal and reject other acoustic activities to be thereafter classified.

**Figure 1:**
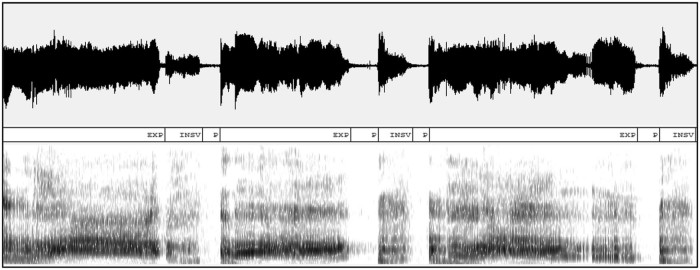
An example of a portion of cry signal with its corresponding components expiration (EXP), audible inspiration (INSV), and pauses (P).

**Figure 2:**
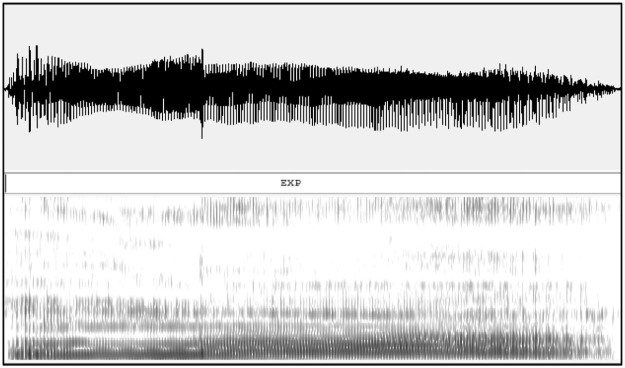
An example of a waveform and spectrogram of an expiration phase.

**Figure 3:**
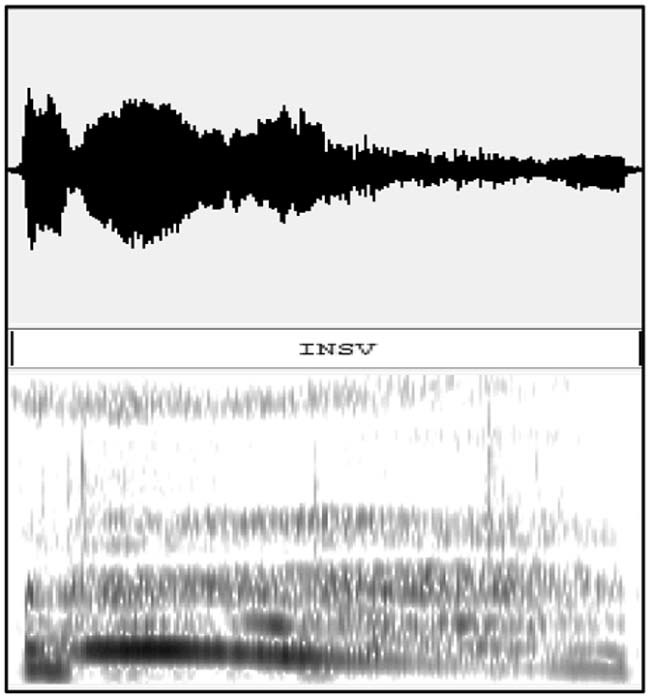
An example of a waveform and spectrogram of an inspiration phase.

Infant cry signals consist of a sequence of audible expiratory and inspiratory phases separated by a period of silence or by unvoiced phases of cry (inaudible expiratory and inspiratory phases during a cry). A cry signal recorded in a real environment usually contains different acoustic activities other than the cry, such as background noise, speech, sound of medical equipment, and silence. This work aims to retrieve most relevant and audible sections from cry signals recorded in a realistic clinical environment, as well as distinction between expiratory and inspiratory phases of the cries. One way to address this problem is to manually segment recorded audio signals and pick out important cry parts. However this manual task is tiresome and prone to errors when the volume of data is large. It is therefore essential to design a segmentation system able to automate this tedious task and be implemented in a real-time clinical decision support tool. Typical waveforms of a cry signal, expiratory phase, and inspiratory phase of cry signals are shown in Figure 1, Figure 2, Figure 3, respectively.

Some attempts to segment cry signals have been reported in the literature. Many studies used the spectrogram to segment cry signals manually through visual and audio monitoring.^5^ On one hand, automatic segmentation is often desired to manipulate all automated diagnostic systems, and on the other hand, because the manual segmentation is an extremely long, tedious task and is prone to errors especially when the amount of data is large. A number of recent works have been done on infant cry segmentation based on the time domain characteristics of the signal. The problem of cry segmentation was being considered as the problem of voice activity detection. Refs. 6, 7 used high-pass filter to reduce most of the background noise, and to distinguish between important and less important parts of the cry signals, they applied short-term energy or/and zero crossing rate by using a satisfactory threshold. However, these methods perform well when cries have been recorded within a laboratory environment and fail under noisy or clinical environment.

In other research efforts, the cry detection problem was considered as the problem of start and end points detection of a cry unit. Based on the hypothesis that cry segments have four times more energy than unvoiced segments, authors in Refs. 8, 9 defined some guidelines to detect cry units based on a dynamic threshold for each record. In these works, authors eliminate not only useless sounds from the signals but also inspiratory sounds of the cry. Another technique used in Ref. 10 considers the problem of cry segmentation as the problem of Voiced/Unvoiced decision. In Ref. 10, authors modified a well-known fundamental frequency estimation method, the Harmonic product spectrum, to check the regularity of the segment analyzed to classify it as an important or not important cry part. In Ref. 11, authors used the simple inverse filter tracking algorithm to detect the voiced frames of cry based on a threshold of the autocorrelation function. The use of threshold limits the attractiveness of the mentioned approaches and decreases their performance in low signal-to-noise ratio levels.

Inspiratory cry parts have been proven to be important in identification of newborns at risk for various health conditions.^12^ Despite this evidence, it is thus surprising that in most research analyzing cry signals, the inspiratory parts of a cry were ignored and not considered in the analysis and the main focus was only on extraction of acoustical data of expiratory parts.

A cry sound segmentation system has been implemented in this work. The proposed system has the capability of detecting three different acoustic classes: audible expiration, audible inspiration, and others (including unimportant acoustics like speech, medical machine sounds, noise, etc). Different signal decomposition techniques such as wavelet packet transform (WPT) and empirical mode decomposition (EMD) have been examined for the features extraction phase.

The WPT has been widely and successfully used in various applications in the voice signal processing domain. It decomposes cry signal into sub-bands to give better resolution. The EMD has been successfully used in denoising and characterizing nonstationary and multicomponent signals such as heart sound signal.^13^, ^14^, ^15^, ^16^, ^17^

Statistic generative models such as Gaussian mixture models (GMM) and hidden Markov models (HMM) have been also chosen as classifiers to distinguish between the three different classes. Recently, GMM and HMM techniques were proven by many researchers to be very successful especially in speaker recognition. These models provide a robust solution when a large amount of training data is available.

The remainder of the paper is organized as follows: the following section is the Recording Procedure and Cry Database section. Then, it is followed by the Proposed Methodology section. Mathematical backgrounds of signal decomposition methods, features extraction, modeling, and classification approaches used in this work are addressed in the Mathematical Background section. An evaluation of the proposed methods and results obtained is reported in the System Evaluation section. Finally, the Conclusion section concludes the paper, offering a list of suggestions for further research.

## Recording Procedure and Cry Database

Data used in this research have been obtained from the newborn Cry-based Diagnostic System (NCDS) database. A description about the data collection technique was presented in a previous work.^18^ A total of 507 cry signals were randomly picked up from the database. Cry signals were recorded with a sampling rate of 44.1 kHz and a sample resolution of 16 bits. The 507 cry signals with an average duration of 90 seconds have been recorded from 203 babies, including both normal and pathological cases.

The constructed dataset contains different kinds of cries, such as pain, hunger, birth cry, etc. It also includes infants' cries in different recording environments and conditions, from silent to very noisy combined with different acoustic activities like speech, machine sounds, noise, silence, etc. Cry signals have been manually segmented and labeled using *WaveSurfer* application (Jonas Beskow and Kare Sjolander in KTH Royal Institute of Technology in Stockholm, Sweden).^19^ Ten-fold cross-validation was carried out to divide the dataset between the training and the testing sets. The dataset was partitioned into 10 folds: nine folds for the training set and the remaining fold for the testing set. Ten tests were conducted with different choice of folds. Data base statistics and details about average time of each class in the testing and training datasets are presented in Table 1, Table 2, respectively.

**Table 1:** Database Statistics

			Number of Babies	Number of Signals
Female	Full term	Healthy	56	141
		Pathological	34	94
	Preterm	Healthy	20	23
		Pathological	17	49
Male	Full term	Healthy	4	11
		Pathological	54	146
	Preterm	Healthy	5	11
		Pathological	13	32
Total			203	507

**Table 2:** Data Used for Training and Testing Corpuses

Classes	Time in Seconds	Average Time for Training Corpus/s	Average Time for Testing Corpus/s
Expiration	21,414	19,348	2,066
Inspiration	2,154.8	1,930	224.8

## Proposed Methodology

**Figure 4:**
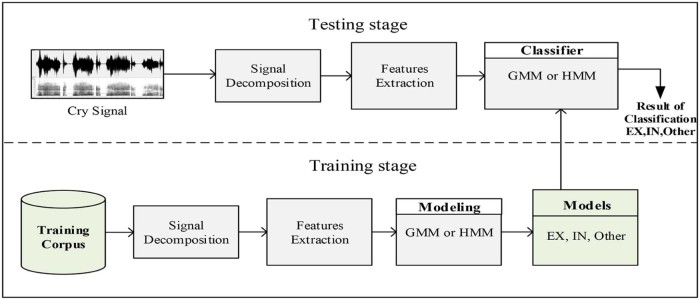
Block diagram of the system architecture.

The basic contribution of this paper is the proposition of a practical cry sounds segmentation system with the ability to detect audible expiratory and inspiratory cry episodes. This section describes the modules required for the development of the proposed system. A block diagram of the general system architecture is presented in Figure 4. The framework is based on supervised pattern classification and it consists of two stages: training stage and testing stage. In either stage, signal decomposition module receives the input cry signal. It converts the original signal from time domain to another domain to better characterize it. Training and testing stages also share the same features extraction module. This module receives the decomposed signal as input and extracts important acoustic information within each frame to form a set of feature vectors. Training involves learning the system and creating an acoustic model for each class based on the acoustic training dataset. Reestimation algorithms are used after the initial training to adapt models' parameters to various conditions. Subsequently, the created models, stored in a database as reference models, are used to classify testing dataset and to measure the system performance during the testing stage. A description of each module is described in the following subsections.

## Mathematical Background

### Signal decomposition

Signal decomposition, also referred to the front-end module in any audio analysis system, is the first step in the proposed method. Because most of the audio signals are nonlinear and nonstationary, a time series and frequency analysis of the signals are needed. Fourier transform, WPT, and EMD are the most common analysis techniques addressed in the literature. In this paper, two cry segmentation systems based on WPT and EMD are designed and compared using the system already designed based on fast Fourier transform (FFT) in our previous work.^18^

#### Wavelet packet transform

**Figure 5:**
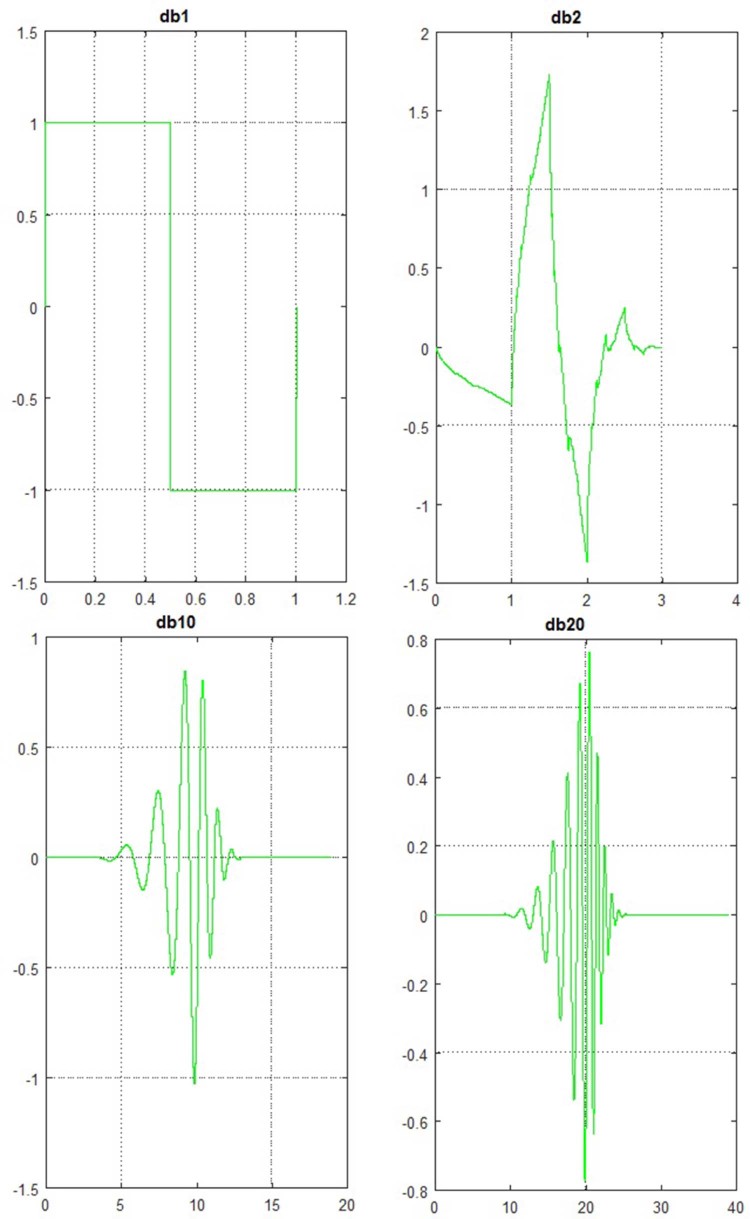
Waveforms of some versions of Daubechies wavelet.

The main objective of the wavelet analysis is to apply varying size of windowing techniques on the signal under study. In lowfrequency band study, a large window size should be used, whereas in high frequency band study, a small window size should be employed.^20^ WPT represents a generalization of wavelet decomposition that could offer a more precise signal analysis by considering both low- and high-pass results. WPT decomposes the original signal into different sub-bands to get better resolution. Each WPT is associated with a level *j*, which splits the frequency band [0, fs/2] to 2*^j^* equal bands by decomposing both low and high frequency components called approximation and detail coefficients, respectively. The result of this decomposition is a balanced tree structure. WPT has been widely and successfully used in various applications in voice signal processing domain. Based on experiences achieved during this work, WPT level 5 on different orders of Daubechies wavelet db1, db10, and db20 is employed in this study. In Figure 5, examples of some wavelet functions from the Daubechies family are shown.

**Figure 6:**
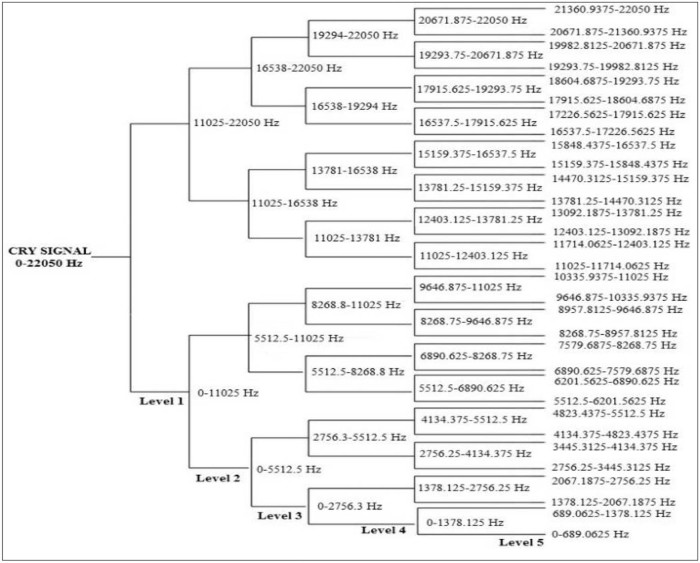
Example of wavelet packet decomposition level 5 of a cry signal at a sampling frequency of 44,100 Hz.

**Figure 7:**
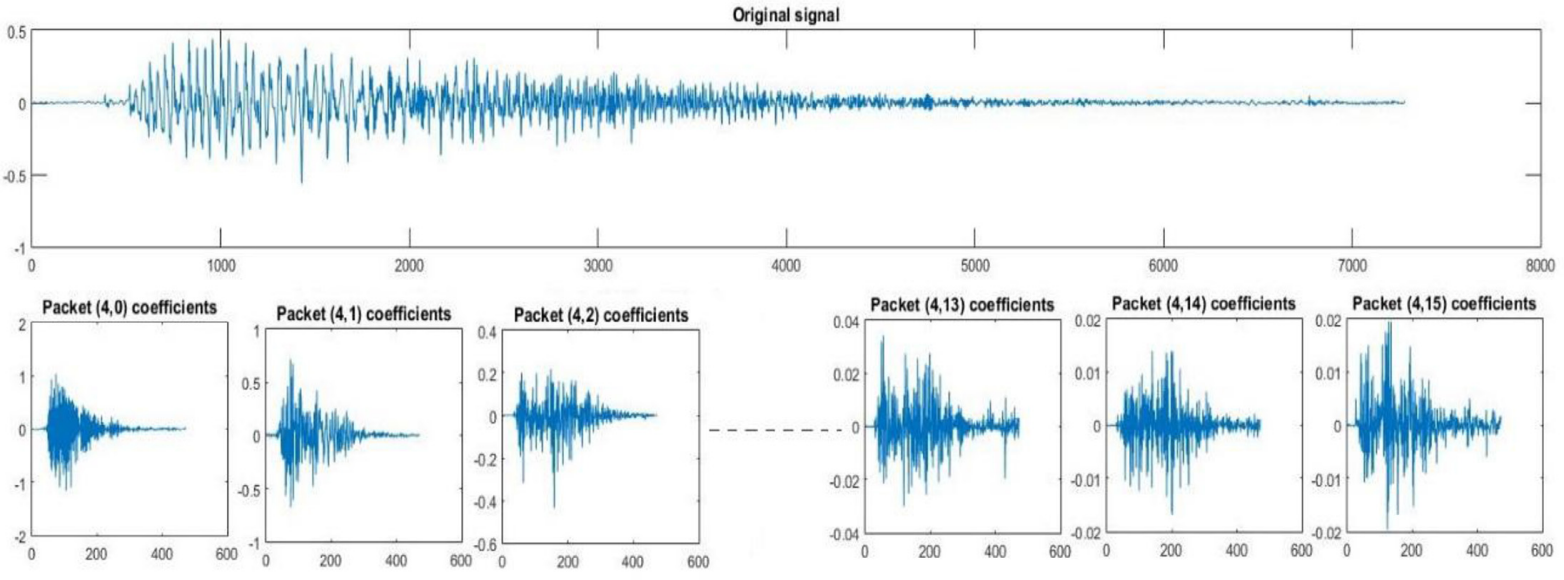
Level 4 of wavelet packet decomposition of an inspiration.

**Figure 8:**
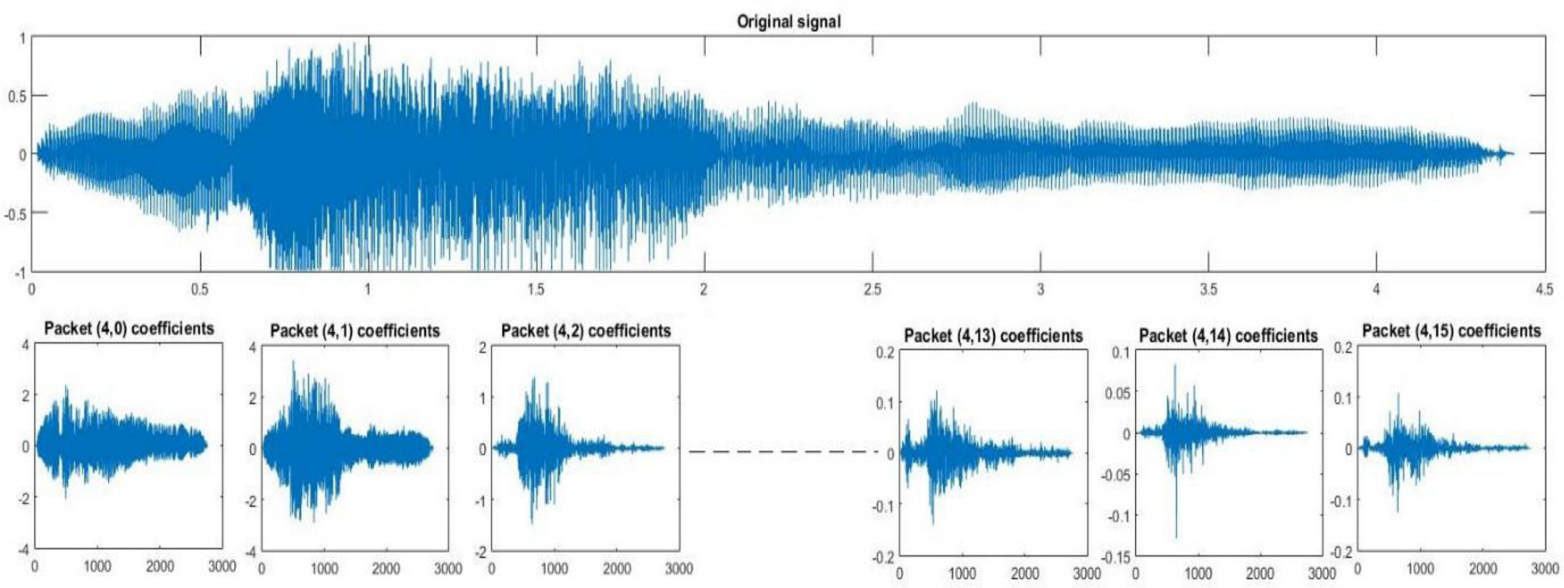
Level 5 of a wavelet packet decomposition of an expiration.

Considering that h(n) is the low-pass filter of length 2N also called scaling filter, and g(n) is the high-pass filter of length 2N also called wavelet filter, wavelet packet functions are estimated using the following equations:

W2n (x)=2∑k=02N−1h(k)Wn (2x−k)W2n+1 (x)=2∑k=02N−1g(k)Wn (2x−k)

where $W_{0}\;\left(x\right)=\varphi \left(x\right)$ is the scaling function and $W_{1}\;\left(x\right)=\Psi \left(x\right)$ is the wavelet function. For more details about wavelet coefficientscalculation, readers are referred to the publication of Mallat.^21^ An example of a wavelet packet tree decomposition of level 5 and the corresponding frequency intervals at each level is given in Figure 6. The sampling frequency used in the present work is 44,100 Hz. Figure 7, Figure 8 are examples of details and approximation coefficients at level 4 of inspiration and expiration phases, respectively.

#### Empirical mode decomposition

The EMD algorithm was proposed by Huang and colleagues in 1998 as an efficient tool to analyze natural signals that are mostly nonlinear and nonstationary. This method decomposes the given signal into a set of functions in time domain and of the same length of the original signal allowing for preservation of the frequency variation in time. This is the key feature of the EMD algorithm that helps to characterize natural signals being produced by various causes at certain time intervals.

**Figure 9:**
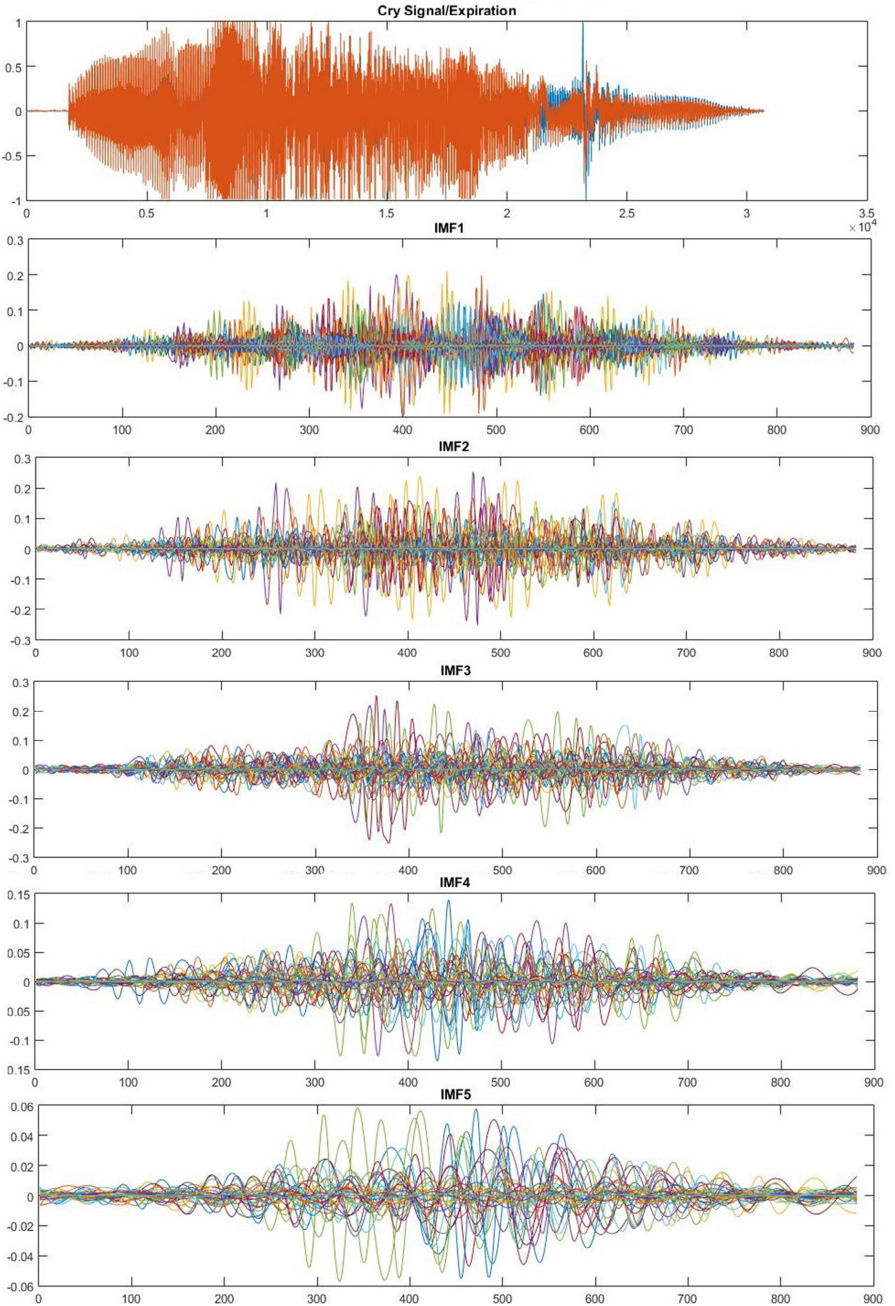
Example of IMF functions of an expiration.

**Figure 10:**
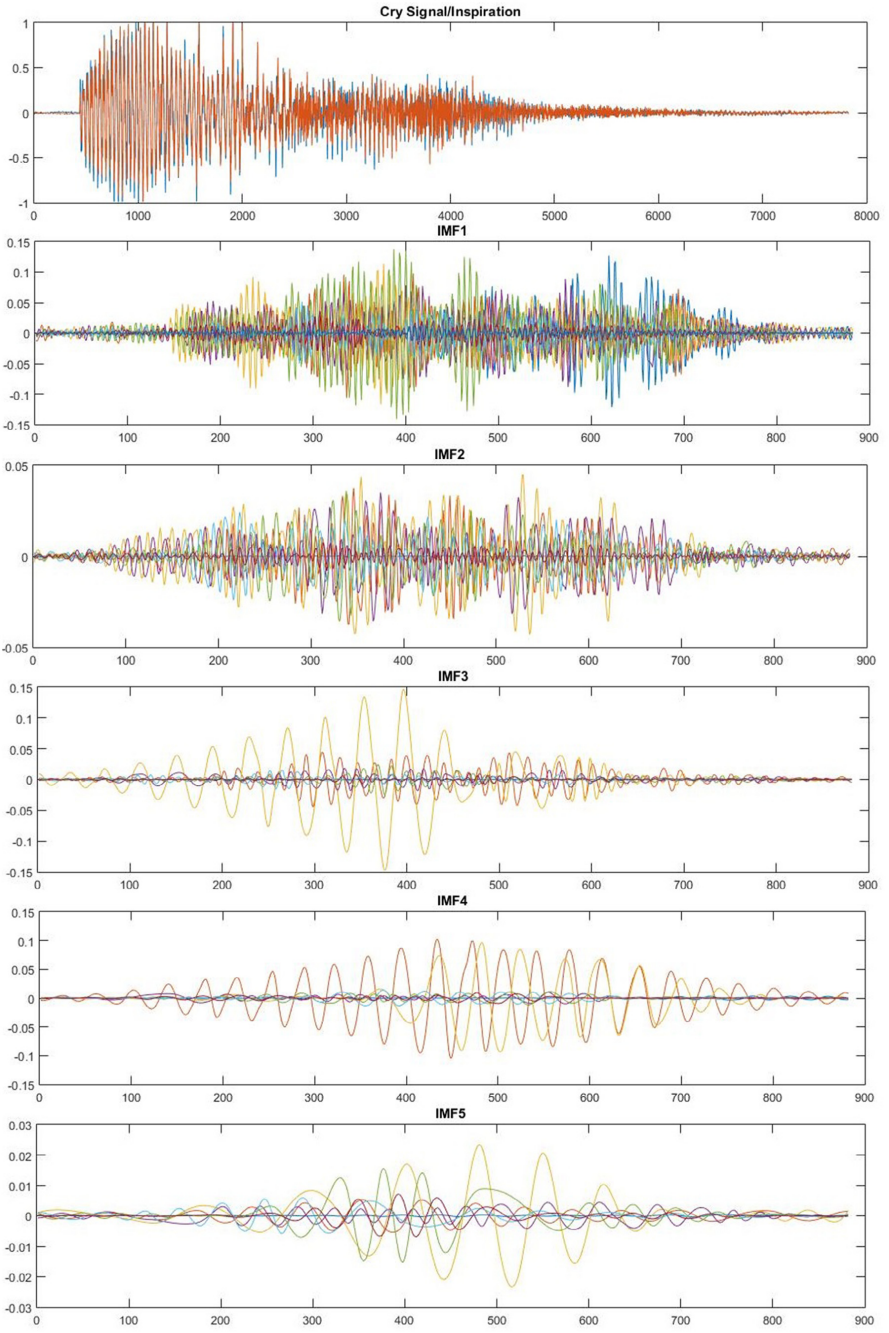
Example of IMF functions of an inspiration.

The EMD algorithm applies a sifting process to break down the given signal into a set of intrinsic mode functions (IMFs), which represents simple oscillatory mode of the original signal. Sifting process is an iterative process during which smooth envelopes are formed by local minima and maxima of the signal, and their mean is subsequently subtracted from the initial signal to finally produce an IMF satisfying two criteria: (1) the number of extremes and the number of zero crossings in the whole sequence of data are equal to or differ by one; (2) the mean value of the envelopes of local extremes is zero at all points. Examples of extracted IMFs from expiratory and inspiratory parts of cry signal using EMD are depicted in Figure 9, Figure 10, respectively.

The following sifting approach has been adopted in this work to extract IMFs from a cry signal x(t):

(1) Identify the local minima and local maxima of the given signal
(2) Interpolate the local maxima using cubic splines interpolation method to form the upper envelope $Env_{U}\;\left(t\right)$
(3) Interpolate the local minima using cubic splines interpolation method to form the lower envelope $Env_{L}\;\left(t\right)$
(4) Obtain the mean envelope of the upper and lower envelopes: $Env_{m}=\frac{Env_{U}\;\left(t\right)+Env_{L}\;\left(t\right)}{2}$
(5) Subtract the mean envelope from the signal: $h\left(t\right)=x\left(t\right)-Env_{m}\;\left(t\right)$
(6) Iterate with x(t) = h(t) until h(t) satisfies the IMF criteria
(7) Calculate the residue by subtracting the obtained IMF from the signal: r(t)=x(t)−h(t)
(8) Repeat the process by considering the residue as the new signal x(t)=r(t) until the termination condition is satisfied.

The original signal can be reconstructed by summing up the obtained IMFs and the residue:

$$x\left(t\right)=\sum_{i=1}^{n}C_{i}\;\left(t\right)+r_{n}\;\left(t\right)$$

where $C_{i}\;\left(t\right)$ and $r_{n}\;\left(t\right)$ represent the i-th IMF and the residue function, respectively. The number of IMFs extracted from the original signal is also represented by n.

The adopted termination condition in this work is the minimum number of extrema in the residue signal. However, usually a certain number of IMFs that contain more important information are used in the next steps. It has been proven through several experiments in this work that the first five IMFs of cry signals have the most important information.

### Features extraction

Features extraction can be defined as the most prominent step in an automatic recognition system. It consists of decreasing the amount of information present in the signal under study by transforming the raw acoustic signal into a compact representation. Among several features extraction techniques that have been used in previous works, Mel-frequency cepstral coefficients (MFCC), which is still one of the best methods, has been chosen. It demonstrates good performance in various applications as it approximates the response of the human auditory system. Wavelet packet–based features have been also chosen owing to their efficiency for segmentation proven in a previous work.^22^

#### FFT-based MFCC

**Figure 11:**
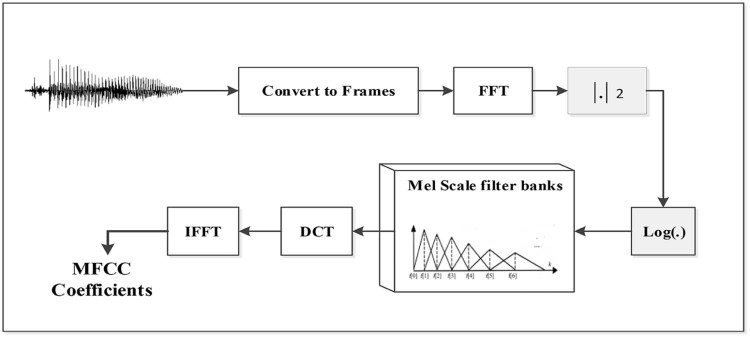
Extraction Mel-frequency cepstral coefficients (MFCC) from the audio recording signals.

MFCCs are used to encode the signal by calculating the short-term power spectrum of the acoustic signal based on the linear cosine transform of the log power spectrum on a nonlinear Mel scale of frequency (Figure 11). Mel scale frequencies are distributed in a linear space in the low frequencies (below 1000 Hz) and in a logarithmic space in the high frequencies (above 1000 Hz).^23^

The steps from original input signal to MFCC coefficients are as follows:

(1) Slice signal into small segments of N samples with an overlapping between segments
(2) Reduce discontinuity between adjacent frames by deploying Hamming window, which has the following form:
  $$w\left(n\right)=0.54-0.46\cos\left(\frac{2\pi n}{N-1}\right),\;0\leq n\leq N-1$$
(3) Use FFT to convert the signal into spectrum form
(4) Consider the log amplitude of the spectrum and apply it to the Mel scale filter banks. The famous formula to convert f Hz into m Mel is given in the equation below:
  $$Mel\left(f\right)=2595\times \log_{10}\;\left(1+\frac{f}{700}\right)$$
(5) Apply discrete cosine transform (DCT) on the Mel log amplitudes
(6) Perform inverse of fast Fourier transform (IFFT) and the resulting amplitudes of the spectrum are MFCCs and are calculated according to the equation below:
  $$c_{n}=\sum_{k=0}^{n-1}\log\left(S_{k}\right)\cos\left[n\left(k-\frac{1}{2}\right)\frac{\pi }{k}\right],\;n=1,\;2,\;\ldots \;,\;K$$
  where $S_{k}$ is the output power spectrum of filters and K is chosen to be 12.

#### Wavelet packet–based features

**Figure 12:**
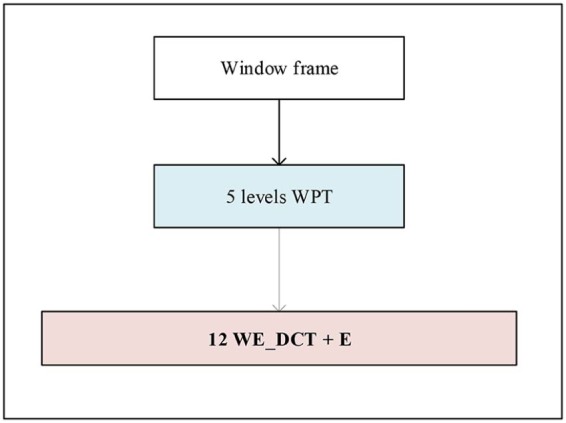
Features extraction step after WPT.

The shortcoming regarding traditional MFCCs is related to the use of FFT whose calculation is based on fixed window size. Another drawback concerning MFCCs is the assumption that the segment is stationary during the frame duration; it is, however, possible that this assumption could be incorrect. To solve this issue, wavelets have been given particular consideration owing to their multiresolution property. The extraction of features based on wavelets similar to MFCC with higher performance has been shown in several works and in different ways.^24^, ^25^, ^26^, ^27^, ^28^, ^29^ In Ref. 26, authors proposed two sets of features called wavelet packet parameters and sub-band–based cepstral parameters based on WPT analysis and proved that these features outperform traditional MFCCs (Figure 12). Authors of Ref. 29 proposed Mel-frequency discrete wavelet coefficients by applying discrete wavelet transform (DWT) instead of DCT to the Mel scale filter banks of the signal. Mel-frequency discrete wavelet coefficient was used in many recent works and proved its performance in speech and speaker recognition.^30^, ^31^, ^32^

In Ref. 23, authors used admissible wavelet packet. The division of frequency axis is performed such that it matches closely the Mel scale bands. In Refs. 32 and 33, another feature extraction technique is presented for deployment with speaker identification: same MFCCs extraction technique presented in FFT-based MFCC section but applied at the input wavelet channels instead of the original signal. In this work, features based on WPT have been considered, and the following steps have been taken for calculation purposes:

The WPT is used to decompose the raw data signal into different resolution levels at a maximum level of j = 5. The normalized energy in each frequency band is calculated according to the formula below:

$$E_{j}=\frac{1}{N_{j}}\sum_{m=1}^{N_{j}}{\left[W_{j}^{n}\;\left(m\right)\right]}^{2},\;j=1,\;2\;\ldots \;,\;B$$

where W _j_^n^ (m) is the m^th^ coefficient of WPT at the specific node W_j_^n^, p is the sub-band frequency index, and B is the total number of frequency bands obtained after WPT.

The Mel scale filter banks are then applied to the magnitude spectrum.

The logarithms of the Mel energies obtained in each frequency band are then de-correlated by applying the discrete cosine transform according to the above formula:

$$\begin{array}{l} WE\text{\_}DCT\left(n\right)=\sum_{p=0}^{B-1}\log_{10}\;\left(S_{p+1}\right)\cos\left[n\left(p+\frac{1}{2}\right)\frac{\pi }{B}\right],\\ \;n=0,\;1,\;\ldots \;,\;B-1 \end{array}$$

WE_DCT stands for wavelet energy–based DCT, which is estimated from wavelet channels and not from the original signal.

#### EMD-based MFCC

These coefficients are estimated by applying MFCC extraction process on each IMF or on the sum of IMFs instead of applying it on the original signal. This technique has been successfully used in many recent works in speech and heart signals classification.^13^, ^14^, ^15^, ^16^, ^17^

**Figure 13:**
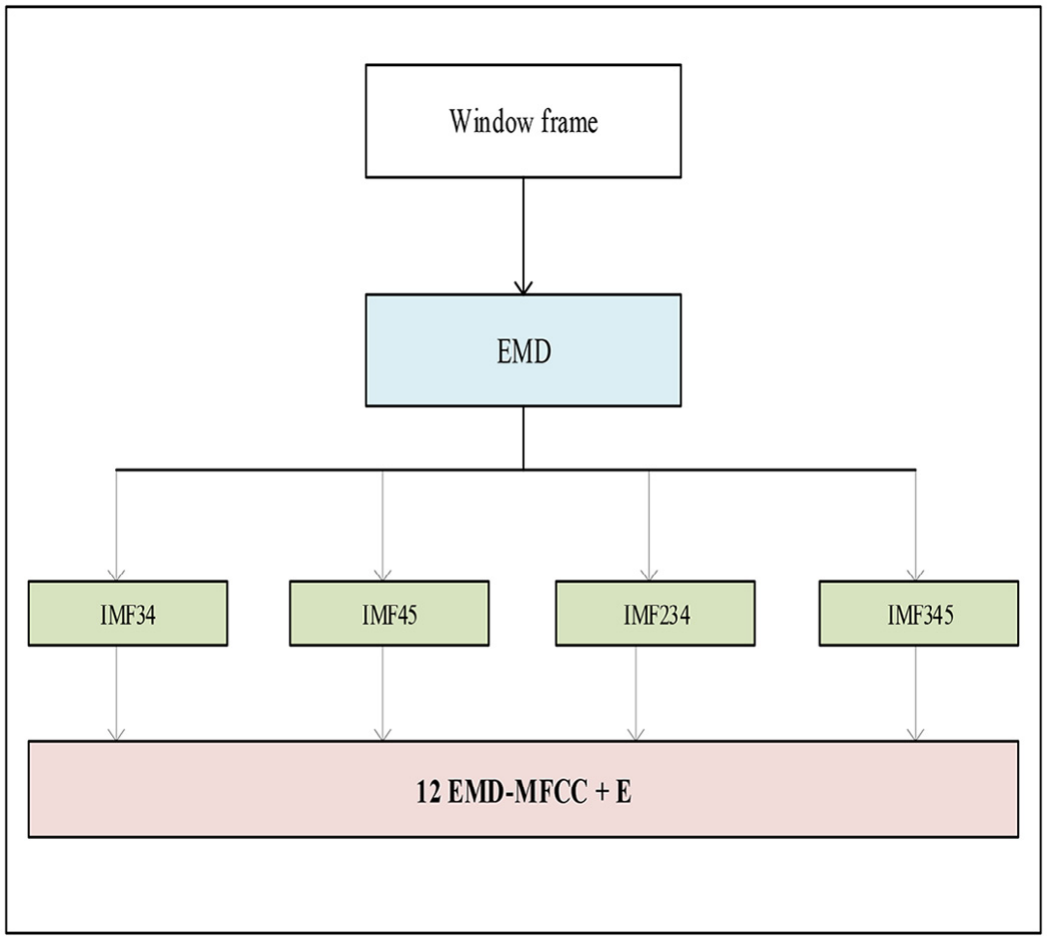
Features extraction step after EMD.

The EMD algorithm with resolution of 50 dB and residual energy of 40 dB has been applied to the subjected cry signals to decompose them into five IMFs (Figure 13). Next, four different combinations of two or three IMFs have been created to be used in feature extraction phase. These sets are as follows:

Set 1: IMF34=IMF3+IMF4
Set 2: IMF45=IMF4+IMF5
Set 3: IMF234=IMF2+IMF3+IMF4
Set 4: IMF345=IMF3+IMF4+IMF5

Twelve Mel-frequency cepstral components as well as their corresponding energy have been further derived from different sets of IMFs.

### Modeling and classification

Once the important parameters are retrieved from an input signal (train or test), these parameters are used as input to a nonlinear classifier whose role is to correctly attribute a class to an input frame under numerous conditions. For the classification stage of this research, two efficient statistical classifiers widely used in machine learning and pattern recognition over the last decades especially in speech and speaker recognition have been chosen: HMMs and GMMs. GMM and HMM are well suited for audio recognition. GMMs are often used owing to their reduced computational costs, whereas HMMs allow a more refined analysis while taking into consideration the variation of the signal over time. In the following subsections, some theoretical backgrounds of these two techniques will be discussed.

#### Gaussian mixture models

The GMM is a probabilistic model for the computation of the probability density function *p* of a set of observed variables *o* using a multivariate Gaussian mixture density. A GMM is represented as a weighted sum of Gaussian distributions and is expressed by the equation below:

$$p\left(o|\lambda \right)=\sum_{j=1}^{J}w_{j}G\left(o:\mu_{j}\text{​},\;\Sigma_{j}\right)$$

where:

p(o|λ) is the likelihood of an input observation *o* of dimension *D*
*J* is the number of mixtures
$w_{j}$ represents positive weighting factors satisfying the constraint $\sum_{j=1}^{J}w_{j}=1$
$G\left(o_{j}\text{​},\;\mu_{j}\text{​},\;\Sigma_{j}\right)$ denotes the jth Gaussian with a mean vector $\mu_{j}$ and covariance matrix $\Sigma_{j}$. It is given by the equation below:
  $$G\left(o;\;\mu_{j}\text{​},\;\Sigma_{j}\right)={\left(2\pi \right)}^{-\frac{D}{2}}\;{\left|\Sigma_{i}\right|}^{-\frac{1}{2}}\exp\left\{-\frac{1}{2}{\left(o-M_{i}\right)}^{T}\sum_{i}^{-1}\left(o-M_{i}\right)\right\}$$

Given a set of observation inputs $\left\{O_{1}\text{​},\;O_{2}\text{​},\;\ldots \;,\;O_{n}\right\}$, GMM has been shown to accurately compute the continuous probability density functions $P=\left\{p_{ij}\right\}$. The parameters of each distribution $w_{j}\text{​},\;\mu_{j}\;and\;\Sigma_{j}$ are estimated by using the expectation-maximization algorithm. Readers seeking more details about GMM should consult the paper of Reynolds and Rose.^35^

During the training stage, and for each audio class defined, the parameters of each Gaussian model are computed from some sequence of training input observations by maximizing the likelihood.

During the classification or testing stage, an observation input is attributed to a specific class for which the likelihood is maximum.

#### Hidden Markov models

HMMs are used in most modern ASR systems. They provide an efficient framework for modeling time-varying spectral feature vectors.^36^ Different applications of HMM in statistical signal processing and acoustic modeling can be found in literature especially in speech and audio domains.^36^, ^37^ An HMM is defined by different sets of parameters: number of hidden states, state transition probability distribution A, observation probability distribution B, and initial state distribution π.

HMM model is denoted as λ = {A, B, π}.

Considering a spectral sequence of observations O = O_1_, O_2_, …,O_T_, one can model the sequence of spectra by using a Markov chain.

$q=\left(q_{0}\text{​},\;q_{1}\text{​},\;\ldots \;q_{t}\right)$ q_t_ as the state of the system at time t, and N as the number of states of HMM.

A=[aij]aij=Pr(qt=j|q=i) 1≤i, j≤N

The probability of q being generated by the Markov chain is given by the following equation:

$$\mathrm{Pr}\left(q|A,\;\pi \right)=\pi_{q_{11}}a_{q_{0}q_{1}}a_{q_{1}q_{12}}\;\ldots \;a_{q_{t-1}q_{t}}$$

For more details about HMM parameters estimation, readers are referred to Refs. 23, 37.

As main training function and to initialize HMM parameters, the Viterbi algorithm is used to find the most likely state sequence for each training input. The log likelihood of the training data is calculated, and the process is repeated until no further increase in likelihood can be found. By applying the so-called Baum-Welch algorithm, the reestimation of the HMM parameters is carried out. The probability of the observation generated by each class is computed to test an unknown observation, and a decision is then taken based on the maximum probability obtained.

## System Evaluation

**Figure 14:**
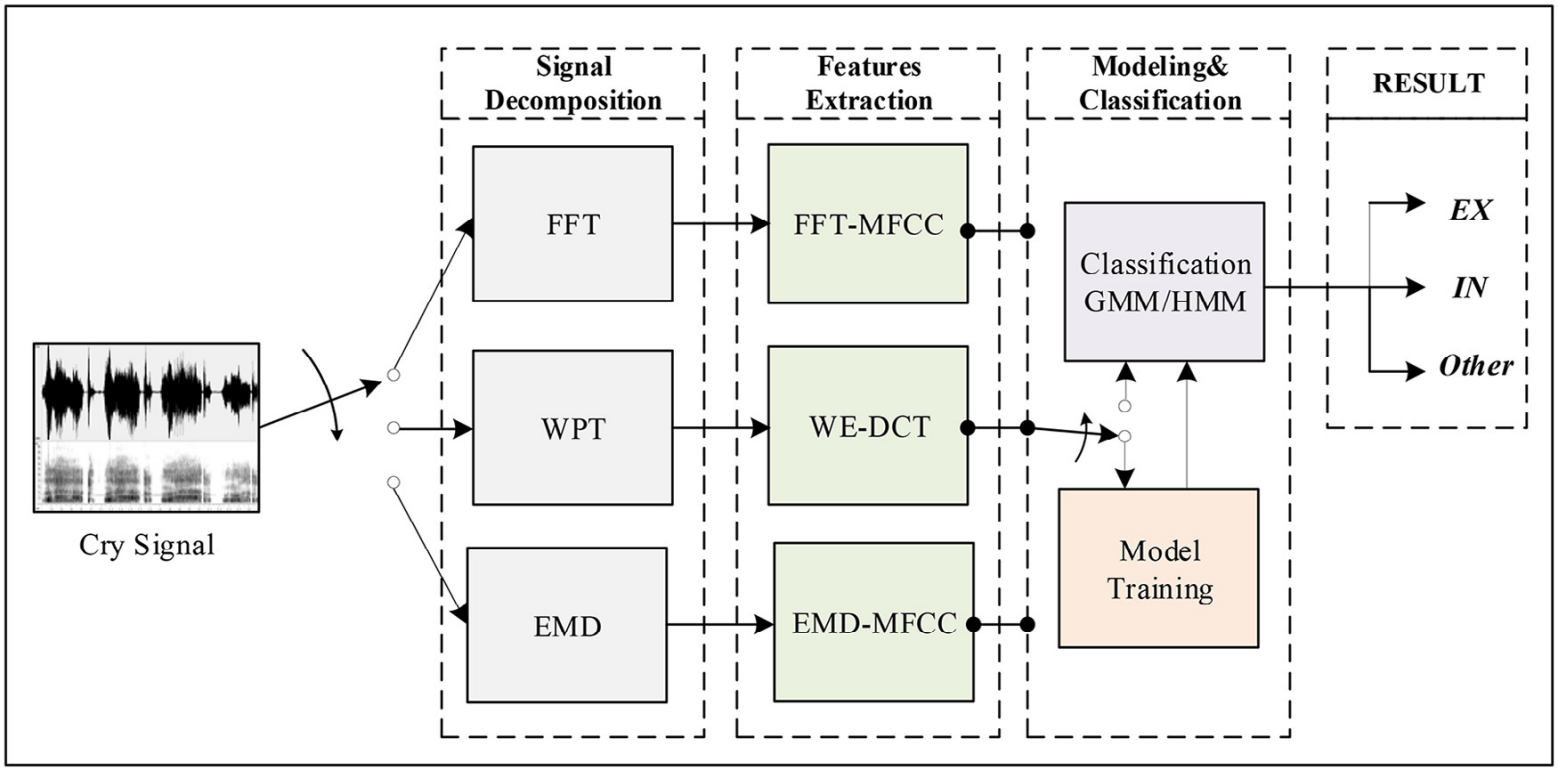
Overview of the different methodologies used in this work.

The aim of this work is to develop an automatic segmentation system with a low error rate. Figure 14 depicts an overview of the adopted methodology. It is based on three essential stages: signal decomposition, features extraction, and classification. In this study, we evaluated the efficiency of nine differently implemented systems listed below by varying approaches in each stage:

(1) FFT+FFT-MFCC+GMM
(2) FFT+FFT-MFCC+4-states HMM
(3) FFT+FFT-MFCC+5-states HMM
(4) WPT+WE-DCT+GMM
(5) WPT+WE-DCT+4-states HMM
(6) WPT+WE-DCT+5-states HMM
(7) EMD+EMD-MFCC+GMM
(8) EMD+EMD-MFCC+4-states HMM
(9) EMD+EMD-MFCC+5-states HMM

GMM-based system is compared with four and five states, left to right HMM-based systems using multiple Gaussian mixtures with diagonal covariance matrices for each class. A varying number of mixtures per state from 16 to 64 Gaussians have been also considered. The efficiencies of the proposed systems are evaluated by comparing their performances with the FFT-based system designed in the previous work.^18^ Each frame was represented by a 13-dimensional feature vector. Two different window frame sizes, 30 ms and 50 ms, with an overlap of 30% are employed.

For both training and evaluation purposes, 507 cry signals used in this paper are manually labeled. The experiments were performed using the 10-fold cross-validation. The whole database is divided several times into two parts: the first part has been used for training and the second part for testing. The average duration of the corpuses used was shown in Table 2. The process of training and testing was repeated for each set of corpuses.To ensure reliable results, the average of the total classification error rate of the same experiments repeated with different sets of training and test corpuses was considered.

To evaluate the efficiency of the systems, the manual transcript files and the files generated at the front end of the system are compared. The performance of the designed systems is then calculated as shown below:

CER=100−Nb of Correctly Classified SegmentsTotal number of Observation in the test Corpus×100%

where CER stands for classification error rate.

Systems 2 and 3 based on FFT decomposition were considered in the previous work.^18^ Training and testing phases using the corpuses described in the Proposed Methodology section are re-executed. Table 3 summarizes the comparison between systems 1, 2, and 3 based on FFT decomposition.

**Table 3:** Classification Error Rates for an FFT-based Extraction Features

FFT_MFCC	30 ms–21 ms	50 ms–35 ms
GMM	8.98	15.99
4-states HMM	26.3	21.23
5-states HMM	23.4	17.29

It can be concluded that based on an FFT decomposition:

(1) a GMM classifier outperforms the four- and five-states HMM classifiers.
(2) a lower window size with GMM classifier gives better results than higher window size.
(3) an HMM classifier produces best results by increasing its number of states.
(4) a higher window size with HMM classifier presents best overall results than lower window size.

**Figure 15:**
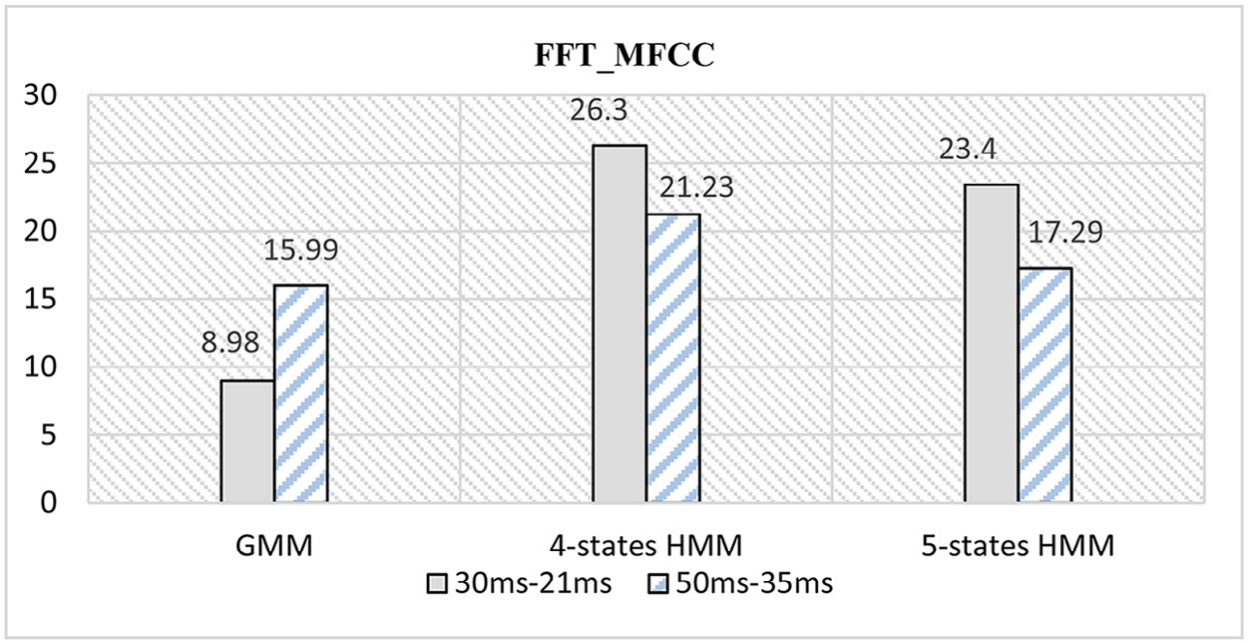
Comparison of CER between different classifiers for an FFT-based MFCC.

The obtained results are summarized in Figure 15. A GMM with 40 mixtures outperforms all experiments and gives a low classification error rate of 8.98%.

Results obtained using systems 4 to 6 are indexed in Table 4 where WPT was employed as decomposition method:

Different levels of decomposition such as 4, 5, and 6 are tried. The best results were obtained using five levels of decomposition. In this paper, therefore, only results obtained by level 5 are addressed.

**Table 4:** Classification Error Rates for a WPT-based Extraction Features

WE_DCT	30 ms–21 ms	50 ms–35 ms
GMM	22.2	29.75
4-states HMM	17.02	27.09
5-states HMM	21.17	27.42

**Figure 16:**
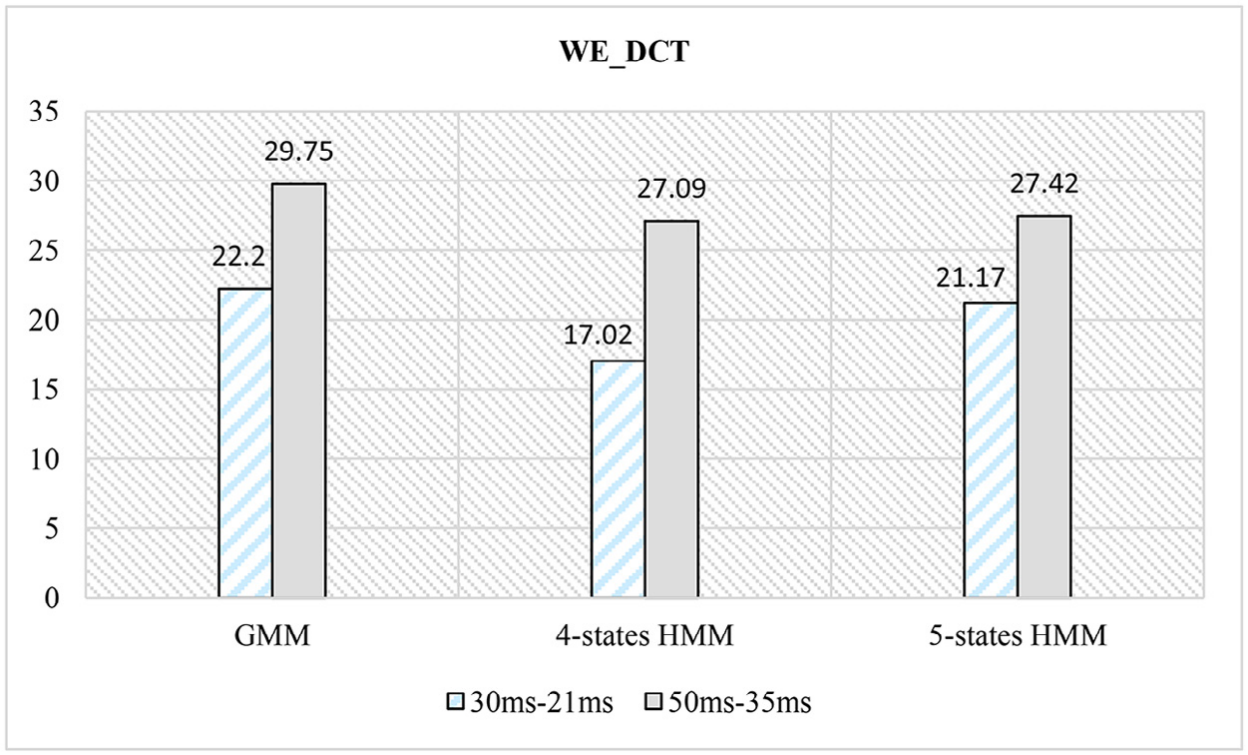
Comparison of CER between different classifiers for a WPT-based features.

From Table 4 and Figure 16, it can be concluded that using a wavelet packet decomposition:

(1) an HMM classifier outperforms a GMM classifier.
(2) a lower window size with either a GMM or an HMM classifier gives better results than higher window size.
(3) an HMM classifier with four states outperforms an HMM classifier with five states.

The results obtained from systems 4, 5, and 6 are shown in the chart in Figure 16. It is proven that lower classification error rate of 17.02% is achieved using a four-states HMM and a window size of 30 ms.

Using the EMD decomposition technique, four sets of different IMF combinations are examined. These four sets are chosen based on results obtained from the experiments of a previous work.^38^

**Figure 17:**
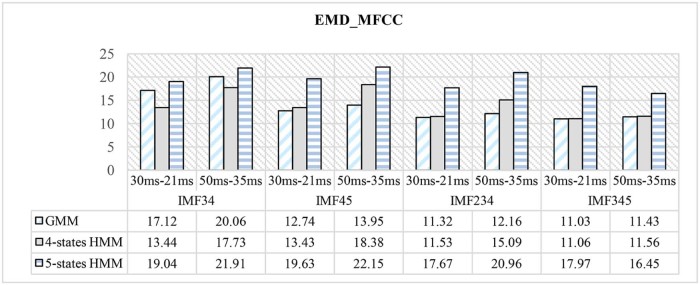
CER of different classifiers used and different window sizes for EMD-based features.

In Figure 17, it can be concluded that while using different combinations of IMFs:

(1) the parameters based on the combination of IMF3, IMF4, and IMF5 yielded the best results.
(2) GMM classifier outperforms an HMM classifier in the set IMF45, IMF234, and IMF345.
(3) four-states HMM outperforms GMM classifier and five-states HMM classifier while using the set IMF34.
(4) best results in most classifiers are obtained using a lower window size.

**Figure 18:**
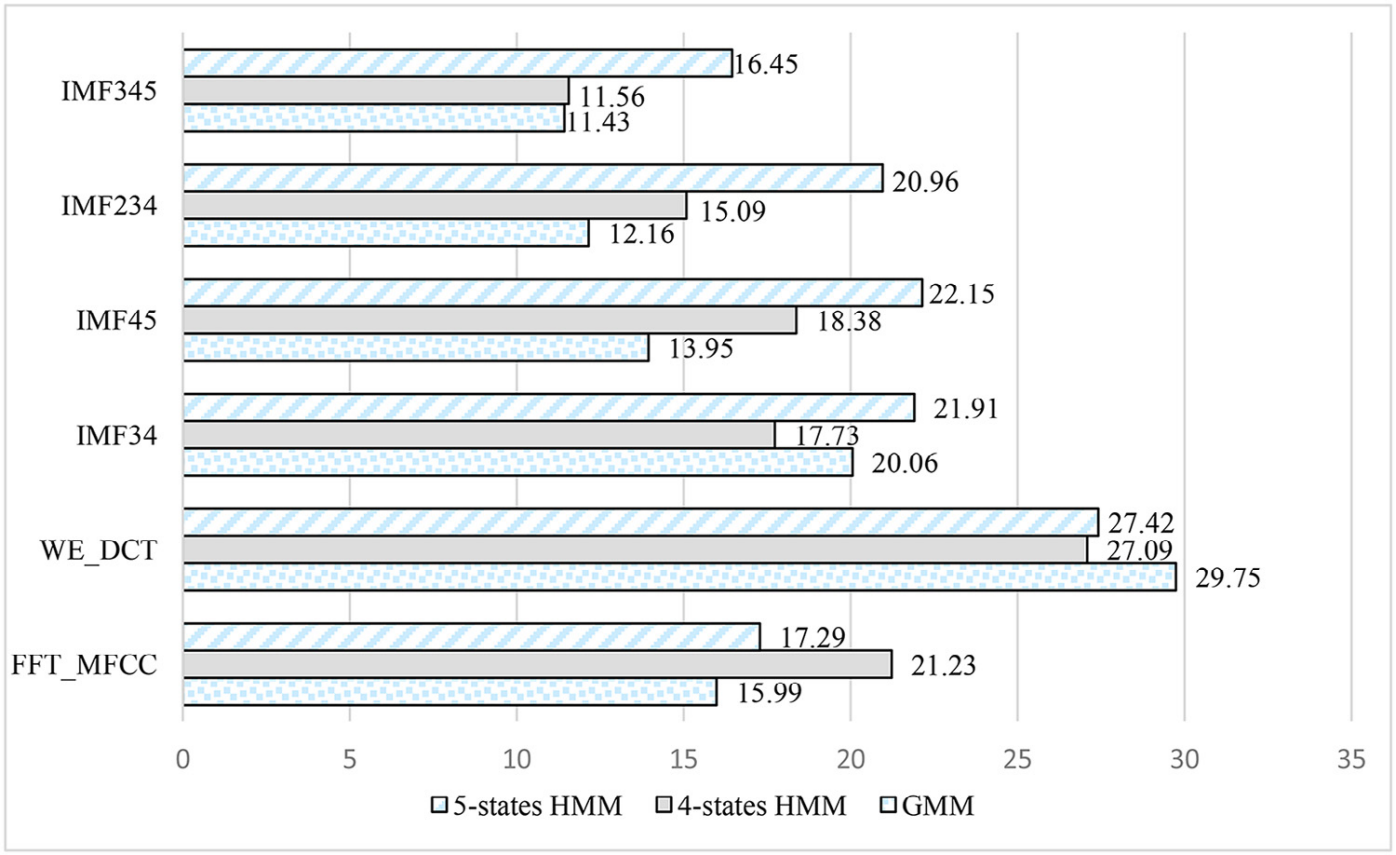
Comparison between CER of different features extracted and different classifiers for a window size of 30 ms.

It can also be seen from Figure 17 that the features represented by the so-called IMF345, which is the combination of IMF3, IMF4, and IMF5, yielded the lowest error rate of 11.03% using again a GMM classifier and a window size of 30 ms. Table 5 and Figure 18 compare the proposed systems in terms of features and classifiers.

**Table 5:** CER of Different Features Extracted and Different Classifiers for a Window Size of 30 ms

Features/Classifier—30 ms	GMM	4-states HMM	5-states HMM
FFT_MFCC	8.98	26.3	23.4
WE_DCT	22.2	17.02	21.17
IMF34	17.12	13.44	17.73
IMF45	12.74	13.43	18.38
IMF234	11.32	11.53	15.09
IMF345	11.03	11.06	11.56

**Figure 19:**
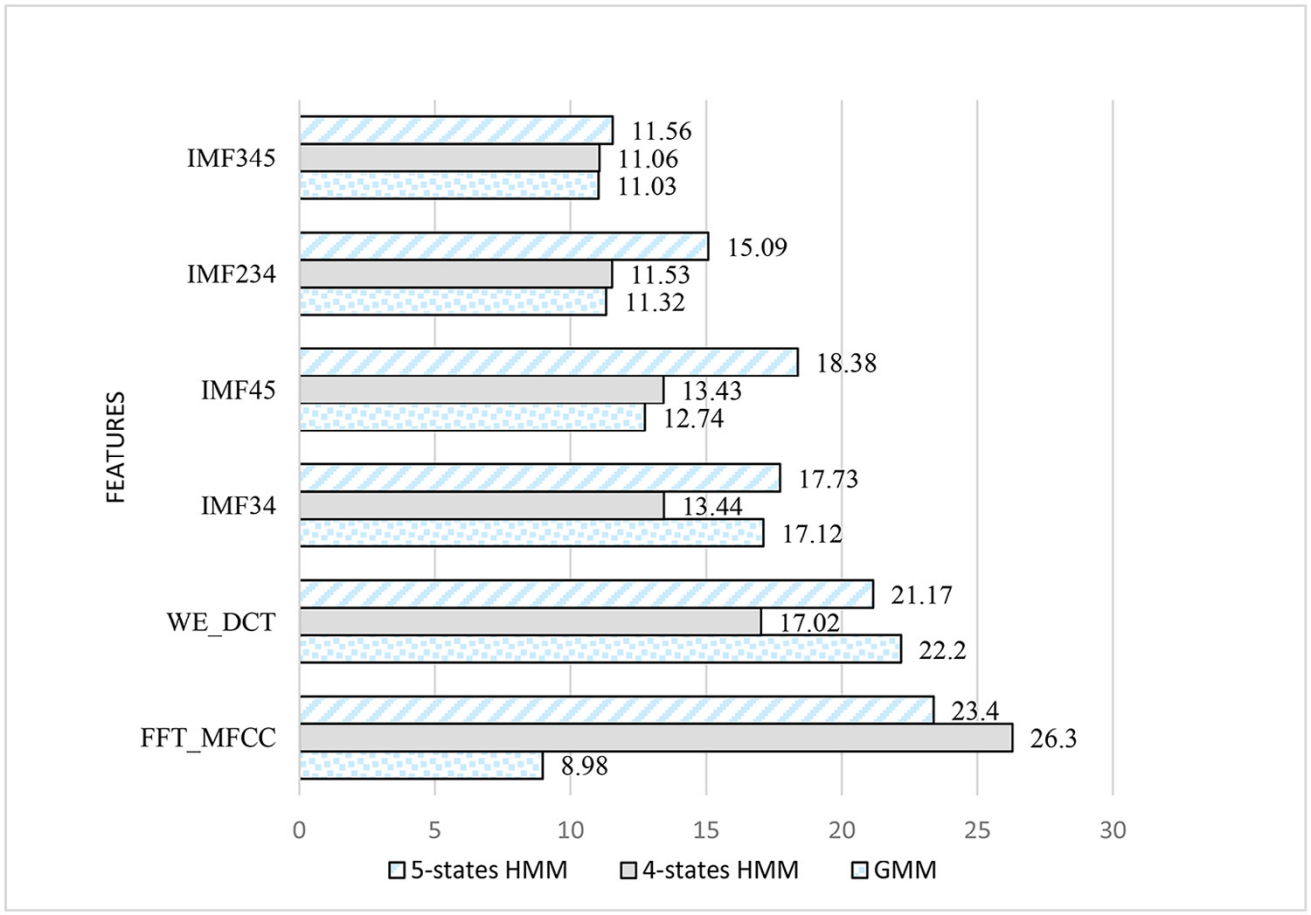
Comparison between CER of different features extracted and different classifiers for a window size of 50 ms.

It can be seen in Table 5 that best results are yielded using the features obtained based on FFT decomposition and using a GMM classifier while using a window size of 30 ms. The minimum obtained classification error rates while employing a 50 ms window size are marked by using EMD decomposition combining IMF3, 4, and 5 and GMM classifier to reach a classification error rate of 11.43%. The results are demonstrated in Table 6 and Figure 19.

**Table 6:** CER of Different Features Extracted and Different Classifiers for a Window Size of 50 ms

Features/4-states HMM—50 ms	GMM	4-states HMM	5-states HMM
FFT_MFCC	15.99	21.23	17.29
WE_DCT	29.75	27.09	27.42
IMF34	20.06	17.73	21.91
IMF45	13.95	18.38	22.15
IMF234	12.16	15.09	20.96
IMF345	11.43	11.56	16.45

To compare the performance of all examined systems in this paper, Table 7 summarizes the best error rate obtained by varying different parameters.

**Table 7:** The Best CER Obtained for the Different Systems Implemented

System	Decomposition Technique	Features Extraction	Classification Method	Best Error Rate %
1	FFT	FFT-MFCC	GMM	8.98
2	FFT	FFT-MFCC	4-states HMM	21.23
3	FFT	FFT-MFCC	5-states HMM	17.29
4	WPT	WE-DCT	GMM	22.2
5	WPT	WE-DCT	4-states HMM	17.02
6	WPT	WE-DCT	5-states HMM	21.17
7	EMD	EMD-MFCC	GMM	11.03
8	EMD	EMD-MFCC	4-states HMM	11.06
9	EMD	EMD-MFCC	5-states HMM	11.56

Analyzing these results, we outline the following conclusions:

(1) System number 1 (FFT+FFT-MFCC+GMM) performed the best among the nine proposed systems by giving an average class error rate of 8.98% for various training and testing datasets.
(2) Next is system number 7 (EMD+EMD-MFCC+GMM) that achieved an error rate of 11.03% by using a combination of IMF345.
(3) It can also be observed that results are the best for the GMM-based classification method in the case of FFT and EMD decompositions and for the four-states HMM in the case of WPT decomposition.
(4) For the FFT decomposition with HMM, best results are reached by increasing the number of states and the window size.

## Conclusion

Newborn cry signals provide valuable diagnostic information concerning their physiological and psychological states. In this paper, EMD-based and wavelet-based architectures have been examined for the purpose of automatic expiratory and inspiratory episodes detection under the scope of designing a complete automatic newborn cry-based diagnostic system. The methodology employed in this research is based on three phases: signal decomposition, features extraction as well as modeling, and classification. Different approaches at each phase have been addressed to implement in total nine different segmentation systems. Three signal decomposition approaches were compared: FFT, wavelet packet decomposition, and EMD. WPT is applied to capture the more prominent features in high and intermediate frequency bands for the segmentation purpose and is compared with IMFs that resulted from EMD decomposition. GMM classifier is also compared with four and five states, left to right HMMs baseline system using multiple Gaussian mixtures with diagonal covariance matrices for each class. Cry signals recorded in various environments are used for training and evaluation of the proposed systems; this dataset includes 507 cry signals with average duration of 90 seconds from 207 babies. To ensure the liability of results, the 10-fold technique is carried out; 90% of the data corpus was randomly chosen for the training stage and the rest 10% for the testing stage while repeating experiments for several times. The effects of different window sizes and different extracted features have been examined. The main goal of this research was to measure the ability of the system to classify audible cries: expiration and inspiration. Results presented in this study show that best results were obtained by using GMM classifier with the low error rate of 8.9%. Future direction of research may include a postprocessing step in the systems designed based on some spectral and temporal approaches to reduce the error rates and increase the performance of the system.
